# Positive Bacterial Culture among Adults with Suspected Urinary Tract Infections Presenting to the Department of Medicine of a Tertiary Care Centre

**DOI:** 10.31729/jnma.8438

**Published:** 2024-02-29

**Authors:** Bishnu Jwarchan, Subash Sapkota, Durga Dhungana, Anil Dhakal

**Affiliations:** 1 Department of Medicine, Manipal College of Medical Sciences, Pokhara, Kaski, Nepal

**Keywords:** *aminoglycosides*, *Escherichia coli*, *prevalence*, *urinary tract infections*

## Abstract

**Introduction:**

Urinary tract infections are the most common infections encountered in clinical practice. Treatment needs to take into account the likely organism, comorbidities and local antibiotic sensitivity pattern. This study aimed to find the prevalence of positive bacterial culture among adults with suspected urinary tract infections presenting to the department of medicine of a tertiary care centre.

**Methods:**

A descriptive cross-sectional study was conducted among adults with suspected urinary tract infections. Data was collected between 1 July 2022 to 31 December 2022 after obtaining ethical approval from the Institutional Review Committee. Individuals with symptomatic urinary tract infections were included in the study. The antibiotic susceptibility tests of the isolates were done. A convenience sampling method was used. The point estimate was calculated at a 95% Confidence Interval.

**Results:**

Among 355 patients, positive cultures were obtained in 148 (41.69%) (36.56-46.82, 95% Confidence Interval). *Escherichia coli* 120 (81.08%) was the predominant organism cultured among the positive bacterial culture cases.

**Conclusions:**

The prevalence of positive bacterial culture was found to be higher than other studies done in similar settings.

## INTRODUCTION

Urinary tract infections (UTIs) are one of the most common reasons for outpatient hospital visits in developing countries.^1^ UTI is found to be more common in diabetics compared to non-diabetic counterparts.^2,3^ Organisms causing UTI include Escherichia coli, Proteus, Klebsiella, Pseudomonas, Streptococcus pneumoniae, Staphylococcus aureus etc.^4,5^

The sensitivity profiles of organisms causing UTIs are different depending on the types of patients and clinical scenarios. When selecting empirical antimicrobial therapy, knowledge of the resistance and susceptibility profile of involved organisms is one of the most important considerations.^6^ Sensitivity and resistance profiles of organisms causing UTI not only differ in different geographical areas but also keep changing over the period. Newer study on these organisms helps us to select proper antibiotics as an empirical therapy in the treatment.

This study aimed to find the prevalence of positive bacterial culture among adults with suspected urinary tract infections presenting to the department of medicine of a tertiary care centre.

## METHODS

This descriptive cross-sectional study was conducted among adults with suspected urinary tract infections presenting to the Department of Medicine of Manipal Teaching Hospital, Pokhara, Kaski, Nepal. Ethical approval was taken from the Institutional Ethical Committee of the same institute (Reference number: MCOMS/IRC/525). Patients aged more than 18 years presenting to the Department of Medicine with clinical features suggestive of UTI and willing to give consent were included in the study. A convenience sampling method was used. The sample size was calculated by using the following formula:


$$\begin{array}{ll} \text{n} & ={\text{Z}}^{2}\times \frac{\text{p}\times \text{q}}{{\text{e}}^{\text{2}}}\\ & =1.96^{2}\times \frac{0.295\times 0.705}{0.05^{2}}\\ & =320 \end{array}$$

Where,

n = minimum required sample size
Z = 1.96 at 95% Confidence Interval (CI)
p = prevalence taken from the previous study, 29.5%^7^
q = i-p
e = margin of error, 5%

The minimum required sample size was 320. However, the final sample size taken was 355.

All patients were advised to give a morning mid-stream urine sample and the collected samples were sent to the microbiology laboratory of the hospitals for culture and sensitivity test. The samples were cultured in fresh Nutrient Agar Media plates and were incubated for a minimum of 24 hours to allow microbial growth. For the identification, morphological examination, gram staining test and biochemical tests were done. Antibiotic susceptibility tests of the isolates were performed on freshly prepared Muller Hinton agar disk diffusion technique. Discs of common antibiotics were placed on each isolate and incubated at 37°C for 24 hours.^8^

Data were entered and analyzed using IBM SPSS Statistics version 16.0. The point estimate was calculated at a 95% CI.

## RESULTS

Among 355 cases, the prevalence of positive bacterial culture was 148 (41.69%) (36.56-46.82 at 95% CI). *Escherichia coii* 120 (81.08%) was the predominant organism cultured among the positive bacterial culture cases (Table 1).

**Table 1:** Bacterial pathogens isolated from the urine specimen (n = 148).

Organism	n (%)
*Escherichia coli*	120 (81.08)
*Proteus*	14 (9.46)
*Klebsiella*	10 (6.76)
*Pseudomonas*	4 (2.70)

Amikacin was found to be 110 (91.67%) sensitive to *Escherichia coli*(Table 2).

**Table 2:** Antibiotic sensitivity pattern (n = 148).

Antibiotic/s	Escherichia coli (n = 120)	Proteus (n = 14)	Klebsiella (n = 10)	Pseudomonas (n = 4)
Amikacin	110 (91.67)	12 (85.71)	8 (80)	4 (100)
Gentamicin	120 (100)	14 (100)	10 (100)	4 (100)
Ceftriaxone	106 (88.33)	12 (85.71)	10 (100)	3 (75)
Cefixime	114 (95)	14 (100)	10 (100)	4 (100)
Norfloxacin	61 (50.83)	8 (57.14)	7 (70)	1 (25)
Ofloxacin	101 (84.17)	11 (78.57)	8 (80)	2 (50)
Nitrofurantoin	88 (73.33)	10 (71.43)	5 (50)	4 (100)

The mean age of the patient was 45.80±20.70 years. A total of 105 (70.95%) were female and 43 (29.05%) were male. Diabetes, fever was present among 43 (29.05%) and 105 (70.95%) respectively. A total of 33 (22.30%) adults with culture positive were admitted.

## DISCUSSION

Among 355 patients, positive cultures were obtained in 148 (41.69%). This was higher than similar studies done in Nepal in Bharatpur (29.50%),^7^ Kanti hospitals (28%),^9^ KIST medical colleges (18.49%)^10^ and Seti zonal hospitals (25.52%).^11^ This is in contrast to the study done in Taiwan which showed 80.30% had positive culture reports.^12^ Studies in Pakistan also showed a high positive rate of 83.90%.^13^ Also study in Ethiopia showed a 90% positive rate.^14^ This difference in the positivity rate is likely due to the timing of the study, duration of the study, patient selection and methods of collection of urine sample, dispatch of the sample, the variation in the equipment used and the manpower involved.

*Escherichia coli* was the most common uropathogen, followed by *Proteus mirabilis, Klebsiella pneumoniae* and *Pseudomonas aeruginosa.* Multiple studies have found Escherichia coli as the most common pathogen also.^7,13-15^ Among the isolated organisms tested for antibiotic sensitivity, it was found that 100 per cent of the isolates were sensitive to gentamicin. This is higher than in studies which showed sensitivity rates ranging from 60% to 94%.^12,13,15^ Amikacin was also found as the second most sensitive antibiotic. Within the tested antibiotics, norfloxacin was found to be the least sensitive one except for *Klebsiella* which had higher resistance to nitrofurantoin. Higher sensitivity for the same antibiotic was reported in another study.^15^ Cephalosporins like ceftriaxone and cefixime did well in the sensitivity testing with 87 and 98 per cent respectively. This was higher than other studies done at multiple centres.^15^ Variations in the antibiotic sensitivity and resistance reports are likely due to the variation in the routine antibiotics, the variation in the empirical antibiotics used for different diseases including urinary tract infection, the antibiotics used for the sensitivity tests and the difference in the patient profiles.

## CONCLUSIONS

The prevalence of positive bacterial culture was found to be higher than other studies done in similar settings. Further research and standardization of methods for urine sample collection, transportation, and antibiotic sensitivity testing, along with continuous surveillance of local antibiotic usage patterns, are crucial to enhance the accurate diagnosis and treatment of urinary tract infections.
